# Pre-existing and machine learning-based models for cardiovascular risk prediction

**DOI:** 10.1038/s41598-021-88257-w

**Published:** 2021-04-26

**Authors:** Sang-Yeong Cho, Sun-Hwa Kim, Si-Hyuck Kang, Kyong Joon Lee, Dongjun Choi, Seungjin Kang, Sang Jun Park, Tackeun Kim, Chang-Hwan Yoon, Tae-Jin Youn, In-Ho Chae

**Affiliations:** 1 Department of Cardiology, Gyeongsang National University School of Medicine and Gyeongsang National University Changwon Hospital, Changwon, Korea; 2 Cardiovascular Center, Internal Medicine, Seoul National University Bundang Hospital, 82, Gumi-Ro 173 Beon-Gil, Bundang-Gu, Seongnam-si, 13620 Gyeonggi-Do Korea; 3 Department of Internal Medicine, Seoul National University, Seoul, Korea; 4 Department of Radiology, Seoul National University Bundang Hospital, Seoul National University College of Medicine, Seongnam-si, Korea; 5 Office of eHealth Research and Businesses, Seoul National University Bundang Hospital, Seongnam-si, Korea; 6 Department of Ophthalmology, Seoul National University Bundang Hospital, Seoul National University College of Medicine, Seongnam-si, Korea; 7 Department of Neurosurgery, Seoul National University Bundang Hospital, Seoul National University College of Medicine, Seongnam-si, Korea

**Keywords:** Preventive medicine, Dyslipidaemias

## Abstract

Predicting the risk of cardiovascular disease is the key to primary prevention. Machine learning has attracted attention in analyzing increasingly large, complex healthcare data. We assessed discrimination and calibration of pre-existing cardiovascular risk prediction models and developed machine learning-based prediction algorithms. This study included 222,998 Korean adults aged 40–79 years, naïve to lipid-lowering therapy, had no history of cardiovascular disease. Pre-existing models showed moderate to good discrimination in predicting future cardiovascular events (C-statistics 0.70–0.80). Pooled cohort equation (PCE) specifically showed C-statistics of 0.738. Among other machine learning models such as logistic regression, treebag, random forest, and adaboost, the neural network model showed the greatest C-statistic (0.751), which was significantly higher than that for PCE. It also showed improved agreement between the predicted risk and observed outcomes (Hosmer–Lemeshow χ^2^ = 86.1, P < 0.001) than PCE for whites did (Hosmer–Lemeshow χ^2^ = 171.1, P < 0.001). Similar improvements were observed for Framingham risk score, systematic coronary risk evaluation, and QRISK3. This study demonstrated that machine learning-based algorithms could improve performance in cardiovascular risk prediction over contemporary cardiovascular risk models in statin-naïve healthy Korean adults without cardiovascular disease. The model can be easily adopted for risk assessment and clinical decision making.

## Introduction

Cardiovascular disease (CVD) is the leading cause of illness and death worldwide^1, 2^. Several risk assessment tools have been proposed to accurately predict the risk of CVD, among which the Framingham risk score (FRS), pooled cohort equation (PCE), systematic coronary risk evaluation (SCORE), and QRISK3 are widely used^3–6^. The individual assessment of cardiovascular risk is a fundamental step for CVD prevention. Contemporary guidelines for primary prevention highly recommend the use of risk calculators to assess the risk of individuals and guide the intensity of preventive interventions^7–10^. However, there is still room for improvement in their accuracy: the area under the curve (AUC) has been shown to be between 0.65 and 0.85^11–13^. In addition, the overestimation of CVD risk, as well as underestimation, have been reported for specific individuals and population subgroups.

Recent years have seen remarkable advances in the application of machine learning (ML) in healthcare and medical research, thanks to high-performance computers^14^. ML is a method of data analysis that automates model building based on patterns and inferences with no prior explicit instructions. The increasing volume and complexity of healthcare information call for the application of big data analytics. ML methods have been increasingly applied in imaging interpretation and shown promising results^15, 16^. They can also be used to develop prediction models from existing data to yield highly accurate results^17^.

This study was designed to assess the calibration and discrimination of pre-existing CVD risk algorithms among Korean adults naïve to cholesterol-lowering therapy. In addition, we developed ML-based risk prediction models and compared their performance with that of contemporary algorithms.

## Related research

Several studies were conducted to verify the pre-existing CVD risk model. The Copenhagen study compared PCE and SCORE^12^. The discrimination function was considered good with C-statistics ranging from 0.71 to 0.85 for PCE and 0.69–0.84 for SCORE. The predicted/observed event ratio was 1.2 for PCE and 5.0 for SCORE, which raises an issue of overestimation. A recent study based on individual-level meta-analysis showed simple recalibration of the pre-existing risk models may help^11^. C-index was shown to range from 0.7010 to 0.7605. To date, only limited number of studies have applied ML techniques for cardiovascular risk prediction in the general population. A study from the Multi-Ethnic Study of Atherosclerosis (MESA) cohort used the random survival forest technique to identify the importance of subclinical disease markers, such as tissue necrosis factor-α receptor, coronary artery calcium score, and carotid ultrasound findings for cardiovascular outcomes^17^. Another study from the MESA cohort utilized support vector machine algorithms, which showed markedly improved discrimination over the PCE model using same parameters of PCE model^18^. Weng et al. also showed improved risk prediction by using ML algorithms from a prospective cohort of 378,256 patients, in which 22 more variables were used in addition to the 8 parameters from PCE^19^.

However, there is still controversy regarding the role of ML for clinical prediction. A meta-analysis of 71 studies demonstrated no definite evidence of superior performance of ML over logistic regression^20^. The authors claimed that model validation procedures are often not sound or not well reported, and that it hampers a fair model comparison. Hot debates followed^21–23^. Despite general optimism about the impact of artificial intelligence, experts think there are still substantial barriers in the real world such as lack of expertise and inadequate regulation^24^.

Cardiovascular risk prediction is one of the fields that improved risk prediction algorithm can benefit the largest population at risk. Conventional cardiovascular risk calculators are basically based on logistic regression. In this study, we tested multiple ML models and sought to evaluate how much they can improve performance. The advantage of the new model was validated using multiple metrics including discrimination, calibration, and decision curve analysis.

## Results

### Characteristics of the study population

**Figure 1:**
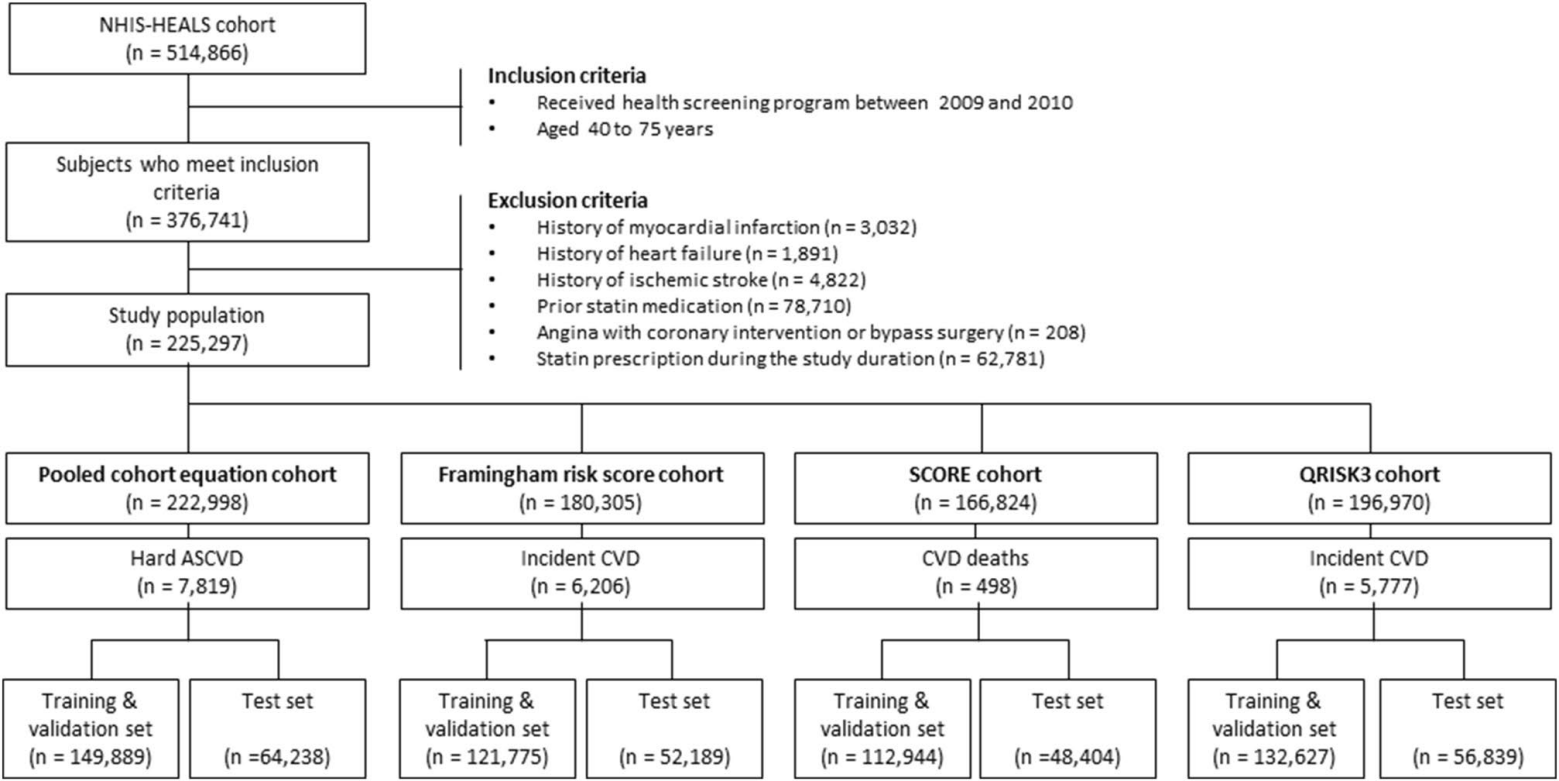
Description of the study population. *NHIS-HEALS cohort* national health insurance service-health screening cohort, *SCORE* systematic coronary risk evaluation, *CVD* cardiovascular disease, *ASCVD* atherosclerotic cardiovascular disease.

The PCE cohort was the main analysis cohort, in which 222,998 individuals with no previous history of atherosclerotic CVD were included (Fig. 1). Their mean age was 58.0 years, 58.1% were men, 5.5% had diabetes mellitus, and 21.1% were receiving antihypertensive treatment (Table 1). During the 5-year follow-up, 7819 subjects experienced atherosclerotic CVD events (event rate: 3.51%) (Supplementary Table S1).

**Table 1:** Baseline profiles of the study population.

Characteristics	PCE cohort (n = 218,299)	FRS cohort (n = 176,271)	SCORE cohort (n = 163,221)	QRISK cohort (n = 196,970)
Common variables				
Age, years	58.0 ± 8.8	56.9 ± 8.3	56.6 ± 8.1	57.6 ± 8.7
Male sex	126,803 (58.1%)	106,418 (60.4%)	97,644 (59.8%)	113,025 (58.7%)
Systolic blood pressure, mmHg	124 ± 15	124 ± 15	124 ± 15	124 ± 15
Total cholesterol, mg/dL	193.3 ± 31.8	193.8 ± 31.8	194.0 ± 31.6	193.6 ± 31.9
HDL cholesterol, mg/dL	55.3 ± 30.3	55.4 ± 29.7	55.2 ± 25.5	55.3 ± 28.9
Antihypertensive medication	46,079 (21.1%)	29,104 (16.5%)	24,815 (15.2%)	37,008 (19.2%)
Diabetes mellitus	12,111 (5.5%)	7420 (4.2%)	N/A	9784 (5.1%)
**Smoking status**				
Non-smoker	177,105 (81.1%)	140,783 (80.0%)	130,489 (79.9%)	155,072 (80.5%)
Current smoker	41,194 (18.9%)	35,488 (20.1%)	32,732 (20.1%)	37,575 (19.5%)
**Variables in QRISK3**				
Body mass index, kg/m^2^	23.6 ± 2.9	23.6 ± 2.8	23.5 ± 2.8	23.6 ± 2.8
Atrial fibrillation	N/A	2088 (1.2%)	1928 (1.2%)	2342 (1.2%)
Chronic kidney disease	17,702 (8.1%)	12,807 (7.3%)	N/A	15,051 (7.8%)
Migraine	14,265 (6.5%)	8412 (4.8%)	8275 (5.1%)	10,775 (5.6%)
Rheumatic arthritis	4241 (1.9%)	2819 (1.6%)	2601 (1.6%)	3491 (1.8%)
Corticosteroid use	8251 (3.8%)	5937 (3.4%)	5595 (3.4%)	6990 (3.6%)
Atypical antipsychotic use	543 (0.2%)	328 (0.2%)	336 (0.2%)	386 (0.2%)
Systemic lupus erythematous	598 (0.3%)	405 (0.2%)	390 (0.2%)	473 (0.2%)
**Smoking status**				
Never smoker	136,236 (62.4%)	106,543 (60.4%)	99,331 (60.9%)	118,987 (60.9%)
Ex-smoker	40,869 (18.7%)	34,240 (19.4%)	29,363 (18.0%)	36,085 (18.7%)
Light smoker (1 ~ 9 pcs)	4557 (2.1%)	3655 (2.1%)	3369 (2.1%)	4051 (2.1%)
Moderate smoker (10 ~ 19 pcs)	15,627 (7.2%)	13,613 (7.7%)	12,508 (7.7%)	14,331 (7.4%)
Heavy smoker (> 20 pcs)	21,013 (9.6%)	18,220 (10.3%)	16,857 (10.3%)	19,193 (10.0%)
**Predicted 10-year risk, %**				
Pooled cohort equations, White	8.7 ± 9.6	7.7 ± 8.5	7.0 ± 7.4	8.3 ± 9.2
Pooled cohort equations, African	9.2 ± 8.0	8.4 ± 7.2	7.7 ± 6.2	8.9 ± 7.8
Framingham risk score	12.7 ± 4.2	12.3 ± 4.0	12.0 ± 3.8	12.5 ± 4.1
SCORE, low	6.7 ± 8.4	6.2 ± 7.8	6.0 ± 7.6	6.6 ± 8.3
SCORE, high	11.9 ± 13.5	11.0 ± 12.7	10.7 ± 12.4	11.6 ± 13.3
QRISK 3	11.3 ± 10.1	10.2 ± 9.2	9.3 ± 8.3	10.9 ± 9.9
**Predicted 5-year risk, %**				
Pooled cohort equations, White	3.7 ± 4.5	3.3 ± 4.0	3.0 ± 3.4	3.6 ± 4.3
Pooled cohort equations, African	5.4 ± 5.4	4.7 ± 4.7	4.3 ± 3.9	5.1 ± 5.2

Data were presented as mean ± SD or % (N).

*FRS* Framingham risk score, *SCORE* systematic coronary risk evaluation, *PCE* pooled cohort equation.

The FRS, SCORE, and QRISK3 cohorts had 180,305, 166,824, and 196,970 individuals, respectively, who matched the target population of each scoring system. Although the risk profiles did not differ largely across the cohorts, there were several distinctions such as no atrial fibrillation in the PCE cohort and no diabetes or chronic kidney disease in the SCORE cohort. Study endpoints were also defined separately in each cohort according to each system. Accordingly, 5-year event rates varied from 0.30% in the SCORE cohort-where only cardiac death was counted to 3.51% in the PCE cohort where hard atherosclerotic CVD was counted.

### Performance of pre-existing risk prediction models

**Figure 2:**
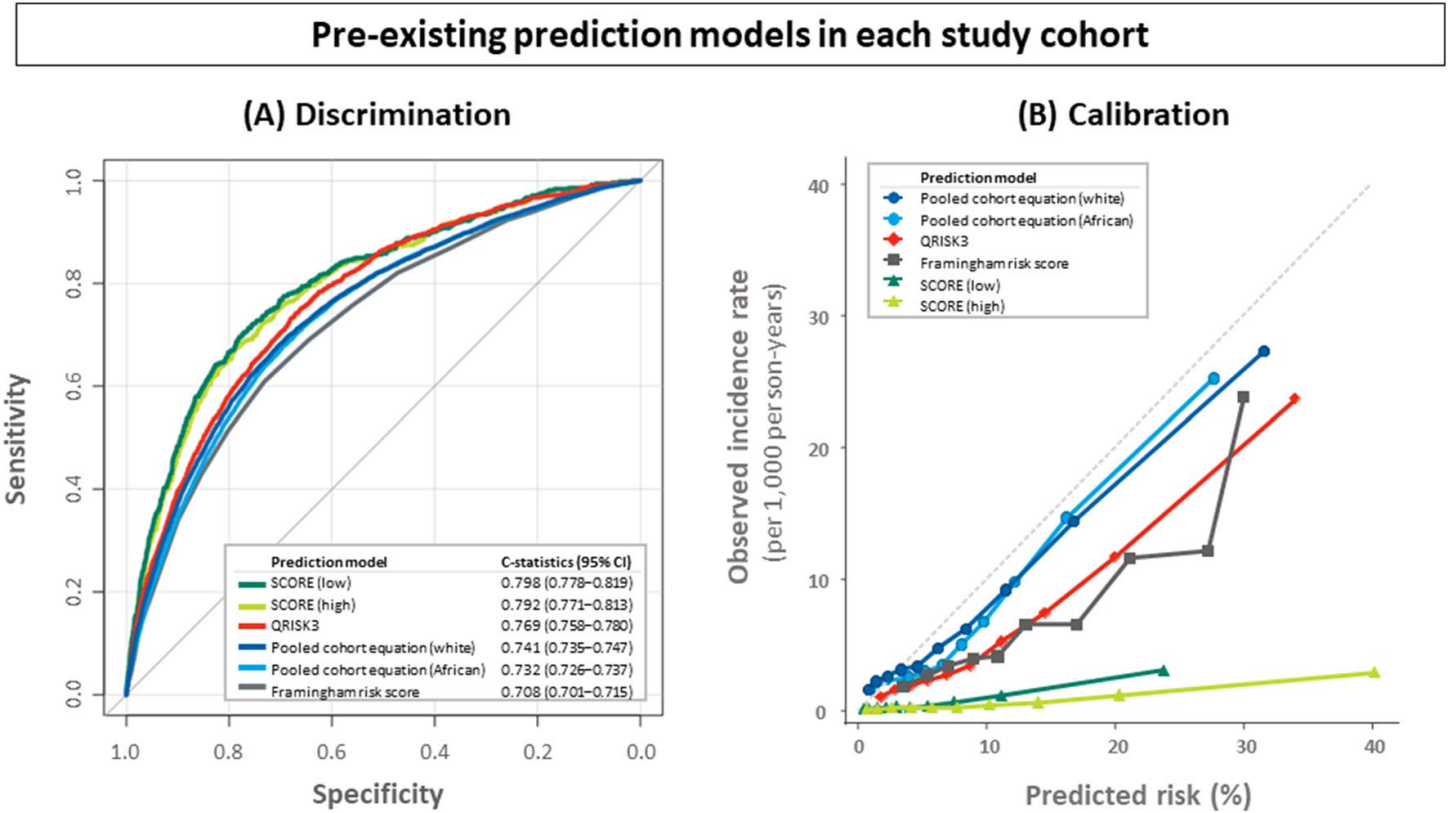
Discrimination and calibration of contemporary prediction models in each cohort. (**A**) Receiver operating characteristic curve analysis for contemporary prediction models. (**B**) Hosmer–Lemeshow calibration plots of contemporary risk prediction models. Risk score-specific predicted (x-axis) and observed events (y-axis) are depicted by deciles of calculated risk. *SCORE (low)* systematic coronary risk evaluation for low cardiovascular disease risk, *SCORE (high)* systematic coronary risk evaluation for high cardiovascular disease risk, *pooled cohort equation (white)* pooled cohort equation for whites, *pooled cohort equation (African)* pooled cohort equation for African Americans.

Figure 2A,B shows the discrimination and calibration of the pre-existing models in each corresponding cohort. All models showed moderate to good discriminatory function with c-statistics between 0.70 and 0.80. In the PCE cohort, the equations for whites outperformed the ones for African Americans (C-statistics [95% confidence intervals (CIs)], 0.741 [0.735–0.747] and 0.732 [0.726–0.737]; p < 0.001). Calibration was plotted for the incidence rate per 1000 person-years against the 10-year predicted risk. PCE showed the best calibration: PCE for whites underestimated the risk in the lower 3 deciles, while overestimation occurred in deciles 7 through 10. FRS, SCORE, and QRISK3 were shown to overestimate the risk compared to the observed incidence rates.

### Performance of machine learning algorithms to the pooled cohort equation cohort

**Figure 3:**
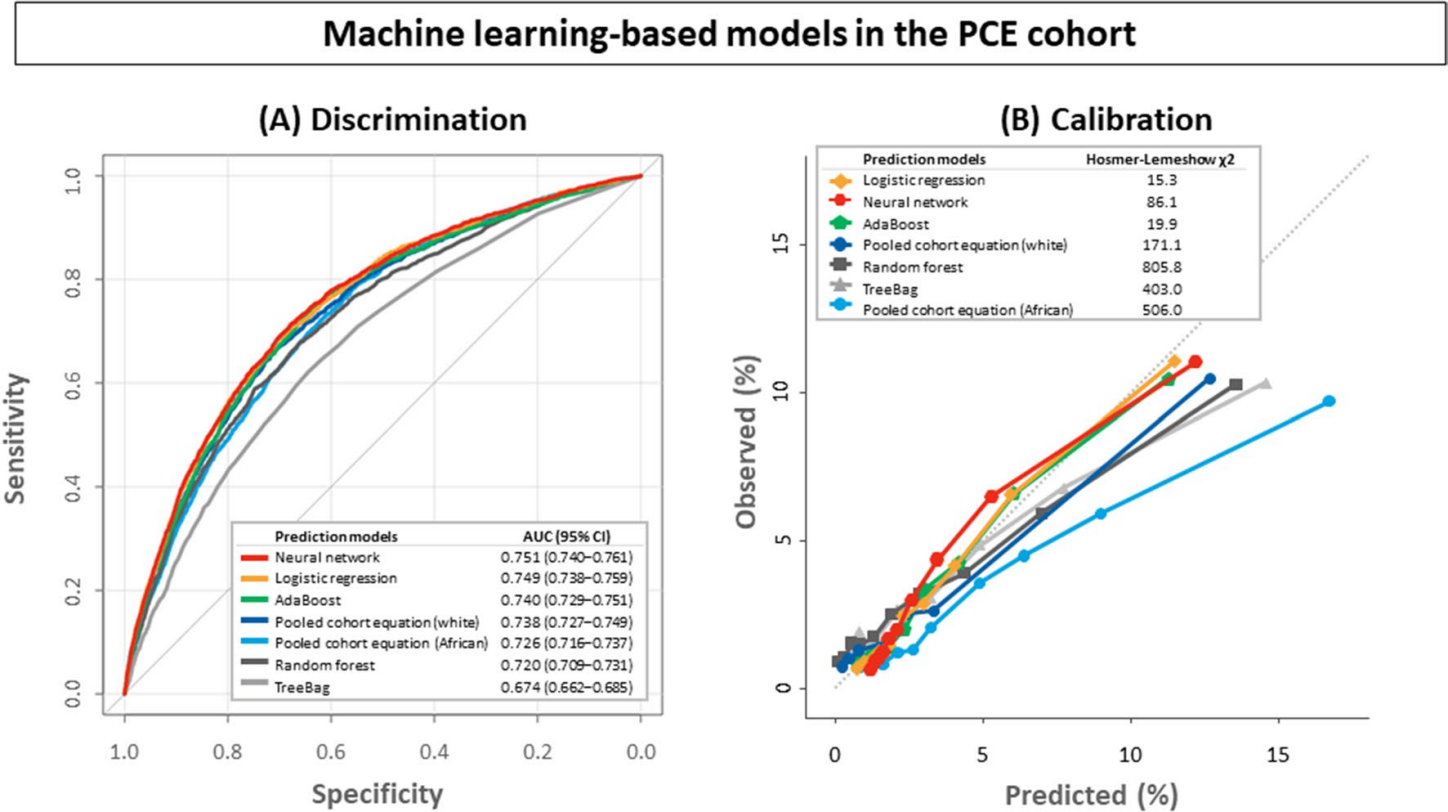
Discrimination and calibration of novel machine learning-based models in the test set of the pooled cohort equation (PCE) cohort. (**A**) Receiver operating characteristic curve analysis and (**B**) Hosmer–Lemeshow calibration plots of PCE and machine learning-based models. Risk score-specific predicted (x-axis) and observed events (y-axis) are depicted by deciles of calculated risk. Pooled cohort equation (white), pooled cohort equation for whites; pooled cohort equation for African Americans.

ML-based algorithms were applied to the PCE cohort. The performance of the ML-based algorithms are detailed in Table 2, and graphically shown in Fig. 3. The Brier score was between 0.030 and 0.032 across PCE and ML-based models. The neural network and logistic regression showed significantly improved discrimination compared to PCE for whites. The neural network exhibited the highest C-statistics (0.751 [95% CIs 0.740‒0.761]), which was significantly greater than that of any other models except logistic regression (Supplementary Table S2). The difference in C-statistics between the neural network and logistic regression was marginal (p = 0.071). A sensitivity analysis was performed with the neural network using 8 variables (age, sex, systolic pressure, total cholesterol, high-density lipoprotein cholesterol, smoking status, history of diabetes, and antihypertensive medication use), which also showed significantly improved discrimination compared to PCE.

**Table 2:** Performance of machine-learning based risk prediction models in the test set of the pooled cohort equation cohort.

Predicted 5-year ASCVD risk	Overall		Discrimination		Calibration		Clinical usefulness	
	Brier	Brier_scaled_	C-statistic (95% CI)	P value	Hosmer–Lemeshow χ^2^	P value	Net benefit at threshold of 3.75%	Net benefit at threshold of 5%
Pooled cohort equation (African)	0.032	35.0%	0.726 (0.716‒0.737)	0.004	506.0	< 0.001	0.0086	0.0049
Pooled cohort equation (white)	0.031	7.3%	0.738 (0.727‒0.749)	–	171.1	< 0.001	0.0106	0.0072
Logistic regression	0.030	4.6%	0.749 (0.738‒0.759)	< 0.001	15.3	0.053	0.0109	0.0079
Random forest	0.031	2.7%	0.720 (0.709‒0.731)	< 0.001	805.8	< 0.001	0.0094	0.0064
TreeBag	0.032	5.9%	0.674 (0.662‒0.685)	< 0.001	403.0	< 0.001	0.0067	0.0038
AdaBoost	0.031	3.9%	0.740 (0.729‒0.751)	0.434	19.9	0.011	0.0107	0.0074
Neural network (16 variables)	0.031	4.4%	0.751 (0.740‒0.761)	< 0.001	86.1	< 0.001	0.0108	0.0078
Neural network (8 variables)	0.031	4.2%	0.748 (0.738‒0.759)	< 0.001	91.2	< 0.001	0.0105	0.0077

*ASCVD* atherosclerotic cardiovascular disease, *CI* confidence interval.

**Figure 4:**
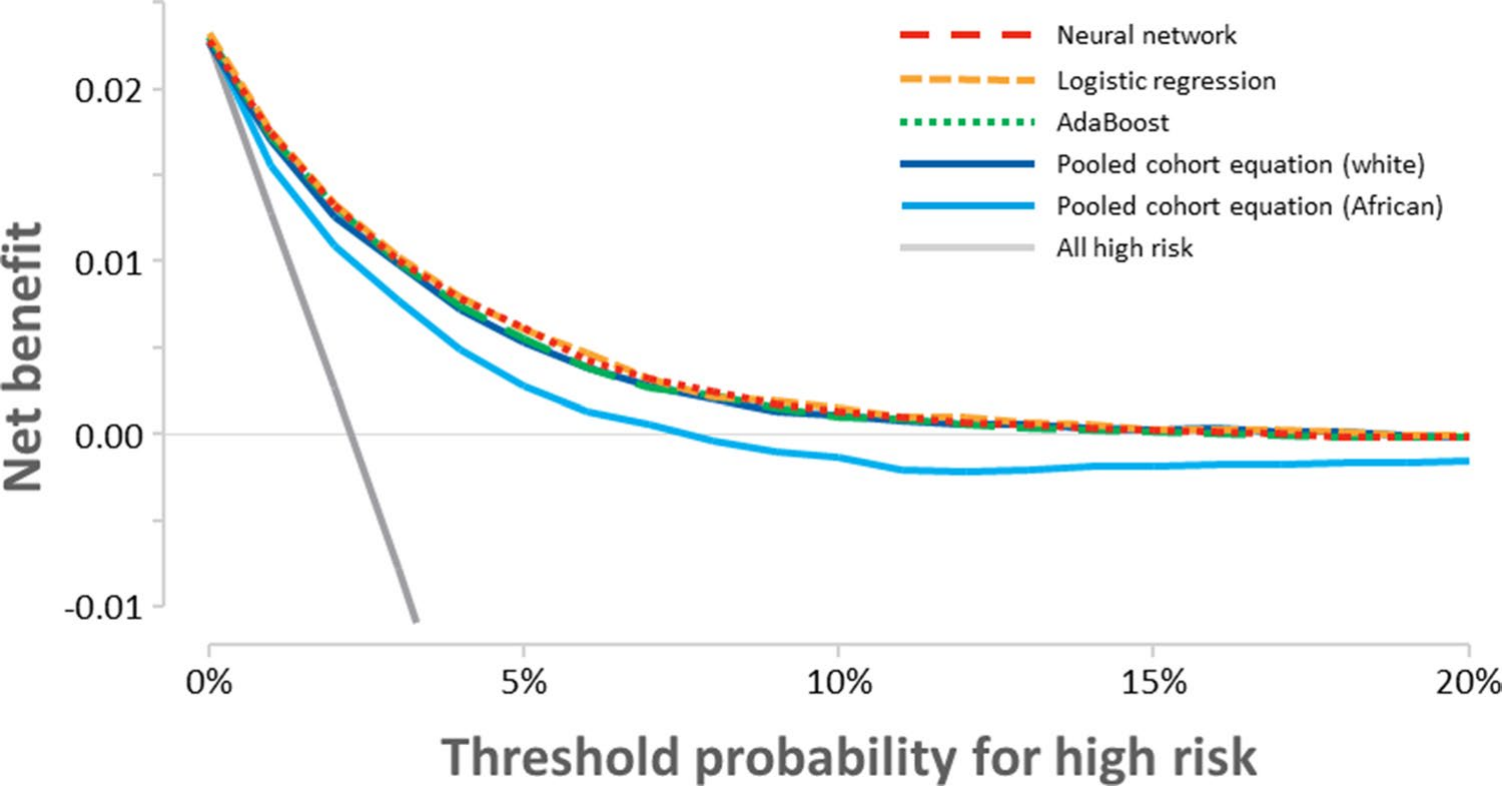
Decision curves for pooled cohort equations and machine learning-based models. Pooled cohort equation (white), pooled cohort equation for whites; pooled cohort equation (African), pooled cohort equation for African Americans.

Calibration was improved with logistic regression, AdaBoost, and the neural network. The Hosmer–Lemeshow χ^2^ values were 171.1, 15.3, 19.9, and 86.1 for PCE for whites, logistic regression, AdaBoost, and the neural network, respectively. Decision-curve analysis showed that ML-based algorithms provided an incremental net benefit across a range of thresholds (Fig. 4). The net benefit values at a threshold of 5% were shown to be 0.0072, 0.0079, 0.0074, and 0.0078 for PCE for whites, logistic regression, AdaBoost, and the neural network, respectively. At this particular cutoff, the neural network-based model would lead to 6 more treatments per 10,000 patients at the same number of unnecessary treatments compared to PCE for whites.

### Performance of machine learning algorithms in other cohorts

Logistic regression and the neural network were also applied to the remaining cohorts (FRS, SCORE, and QRISK3 cohorts) (Supplementary Table S3). Logistic regression and the neural network showed significantly higher C-statistics than FRS, and logistic regression showed significantly higher C-statistics than SCORE. No ML algorithms outperformed the pre-existing prediction model in the QRISK3 cohort.

## Discussion

In this study, we found that pre-existing risk models showed acceptable performance in predicting cardiovascular risk in real-world Korean adults who were free from CVD and naïve to statin therapy. However, they were mostly shown to overestimate individual risk and to have moderate to good discrimination. On the other hand, models using ML techniques were shown to improve cardiovascular risk prediction. Algorithms using logistic regression, AdaBoost, and the neural network showed significantly higher discrimination and better calibration than pre-existing calculators.

Prevention is the most effective way to reduce the impact of CVD^25^. Current guidelines recommend that the assessment of CVD risk should be the start of cardiovascular risk-reducing strategies. The Third Report of the Expert Panel on Detection, Evaluation, and Treatment of High Blood Cholesterol in Adults guidelines recommended the use of the FRS^26^. European guidelines recommend risk assessment via the SCORE system^9, 10^, the United States’ guidelines advocate for the PCE^4, 7^, and QRISK has been endorsed by the National Institute for Health and Clinical Excellence in the United Kingdom. Risk prediction is considered the key component in deciding treatment strategies. The American College of Cardiology /American Heart Association (ACC/AHA) guidelines for high blood pressure recommend medical treatment for primary prevention if a patient with hypertension (defined as ≥ 130/80 mm Hg) has an estimated 10-year atherosclerotic CVD risk of ≥ 10%^8^. Similarly, statin therapy should be considered in adults with a 10-year atherosclerotic CVD risk of ≥ 7.5% according to the ACC/AHA guidelines on blood cholesterol^7^.

The performance of risk prediction models has been validated by a number of studies^11–13, 27, 28^. Similarly, our study demonstrated the competency of risk prediction algorithms in the real world. All pre-existing models showed C-statistics of 0.70‒0.80 for their dedicated endpoints. PCE showed relatively good agreement between the predicted risk and observed event rates, while FRS, SCORE, and QRISK3 were shown to overestimate risk in this study population. Previous studies on the Korean population have also shown that the accuracy of preexisting models was fairly good^29, 30^.

Our study showed that several ML techniques including the neural network led to improved cardiovascular risk discrimination and calibration as well as net benefit. The AUC of the neural network was + 0.13 compared to that of PCE for whites while calibration was significantly better. In addition, the improved performance also resulted in net clinical benefit: better classifying the patients who require blood pressure-lowering or lipid-lowering therapy. An artificial neural network solves a problem through the learning process by controlling the strengths of connections between complexly intertwined neurons. The learning process is similar to human learning, memory, and inference. Its advantages include identifying arbitrary nonlinear multiparametric discriminant functions. In this manner, neural networks enable the learning of highly complex functions and accurate predictions for complex decision-making problems^31^.

Although ML-based models were shown to have better prediction capabilities, there may be criticism regarding the performance of ML-based algorithms. Firstly, ML-based algorithms typically use large numbers of variables, some of which are not routinely recorded in clinical practice. Conventional risk prediction models have been developed to be broadly used cost-effectively, and therefore, use only a small number of essential variables. However, our sensitivity analysis showed that even after limiting the number of variables, ML-based algorithms still showed better performance than conventional models. Secondly, although there was an improvement, the absolute degree of improvement was small. The neural network model showed significantly increased C-statistics compared to PCE, but the absolute increase was no greater than + 1.3%. Although statistically significant, it is reasonable to assume that this was only a modest improvement. However, ML, especially the artificial neural network, is expected to provide better data interpretation and risk prediction as the volume of medical information exponentially increases.

This study has several limitations. Firstly, only 5-year follow-up data were available in the present study. Most risk prediction models aim to predict 10-year outcomes. However, the use of population-based data allowed for a large sample of statin-naïve healthy adults without CVD. Most contemporary prospective studies are not free from potential bias associated with statin use, which may cause an effect modification. Secondly, the study is not free from selection bias since the study population was chosen from the recipients of the general health screening program. However, the national insurance system covers 97% of Korean residents. The health screening program included 51.2% of the recipients in 2009 and 54.1% in 2010 according to the national statistics^32^. Thirdly, there is a potential risk of misclassification bias as many covariates and outcomes were defined using claims information^33^. For example, the status of blood pressure-lowering treatment may have changed during the follow-up duration, which was not considered in the model.

Pre-existing risk prediction models, such as the FRS, SCORE, PCE, and QRISK3, showed good performance in statin-naïve healthy Korean adults without CVD. This study suggests that ML-based cardiovascular risk prediction algorithms offer improved discrimination and calibration over contemporary models. Future studies are required to test the feasibility and usefulness of our models in the real-world clinical practice.

## Methods

The data reported in this article are available to other researchers via application to the National Health Insurance Sharing Service (https://nhiss.nhis.or.kr/) for purposes of reproducing the results or replicating the procedure.

### Data source and study individuals

The study subjects were extracted from the National Health Insurance Service-Health Screening (NHIS-HEALS) cohort from Korea. The cohort design and profiles have been reported previously^34^. In brief, the insurance system covers 97% of Korean residents. General health screening programs are provided to all insured adults aged 40 years or older every 2 years for the prevention and early detection of major diseases. The National Health Insurance Service-Health Screening cohort includes 514,866 individuals who participated in health screening programs from 2002 to 2015.

Individuals who participated in the health screening program between 2009 and 2010 were chosen for this study. This selection of time period was to ensure a complete 5-year follow-up because the screening program started including fasting serum lipid levels (total cholesterol, triglycerides, high-density lipoprotein cholesterol, and low-density lipoprotein cholesterol) in 2009. Follow-up data until December 2015 are provided for the cohort. In line with the target population of contemporary scoring systems, selection criteria included (1) age between 40 and 79 years, (2) no previous diagnosis of CVDs, such as myocardial infarction, ischemic stroke, and congestive heart failure, (3) Those with angina who received coronary revascularization therapy, such as percutaneous coronary intervention and coronary artery bypass surgery were excluded. (4) In addition, to avoid bias caused by statin therapy, individuals who had been receiving a statin before the screening or started statin therapy during the study period before the obtaining of the study outcomes were also excluded.

Next, 4 separate cohorts were built following the intended target population and outcome definitions of each scoring system: the FRS, PCE, SCORE, and QRISK3 cohorts (Fig. 3). The definitions of the cohort population and study outcomes are detailed in Supplementary Table S4. The PCE cohort was the main target of analysis and results from the FRS, SCORE, and QRISK3 cohorts were provided for sensitivity analyses. The Seoul National University Bundang Hospital’s institutional review board determined that our study was exempt from review (X-1708-417-911). The present study was performed in accordance with the Declaration of Helsinki and the need for informed consent was waived.

### Risk factor variables and risk score calculations

Sixteen variables were selected as risk factors: 8 variables that were commonly used in the established risk prediction models, and 8 variables used in only QRISK3. The 8 common variables included age, sex, systolic blood pressure, total cholesterol, high-density lipoprotein cholesterol, smoking status, history of diabetes, and antihypertensive medication use. Demographic characteristics such as age and sex were extracted from the enrolment status database. Systolic blood pressure, total cholesterol level, and high-density lipoprotein cholesterol level were derived from the results of the health screening program. Smoking status and the amount of smoking were identified using self-report questionnaires. Histories of diabetes and hypertension medication use were identified using previous claims data from 2002 until the date of enrollment. The 8 variables from the QRISK3 algorithm were steroid use, body mass index (kg/m^2^), atrial fibrillation/flutter, migraine, systemic lupus erythematosus, rheumatic arthritis, atypical antipsychotic use, and chronic kidney disease (Supplementary Table S5). Erectile dysfunction and schizophrenia, which are also used in the QRISK3 algorithm, were not included in this study because as the accuracy of the former has not been validated and the latter was not available from the NHIS-HEALS cohort due to privacy issues. No imputations were applied for continuous variables (age, systolic blood pressure, total cholesterol, high-density lipoprotein cholesterol, and body mass index), and subjects with any missing values and outliers were removed from the cohort.

Four types risk prediction scores were calculated with equation-based methods using patients’ baseline data: the FRS, PCE, SCORE, and QRISK3 (Supplementary Table S4)^3–6^. PCE was originally developed to obtain 10-year cardiovascular risk. The predicted risk at 5 years was calculated using parameters that were published previously by Muntner et al.^27^ Because Asian ethnicity is not represented in the PCE, both the equations (one for whites and the other for African-Americans) were calculated. Similarly, two risk calculators of the SCORE (one for low-risk populations, and the other for high-risk populations) were studied.

### Outcome

The study endpoints were defined separately in each cohort following the definitions of each algorithm (Supplementary Table S5). The PCE cohort was the main study cohort, where the endpoint was first hard atherosclerotic CVD (defined as cardiac death, non-fatal myocardial infarction, and fatal or nonfatal stroke). Mortality was determined from the National Death Index by linking identification codes to the corresponding individual. Cardiac death was defined as death due to cardiovascular etiology. Nonfatal myocardial infarction and ischemic stroke were determined with claims records. Myocardial infarction was defined by discharge diagnosis codes I21 and I23 of the International Classification of Diseases, 10th Revision (ICD‐10). Stroke was defined as a discharge diagnosis (ICD-10-code, I63) of patients who needed hospitalization and underwent brain imaging, such as computed tomography and magnetic resonance imaging. Individuals were followed up until death from any cause or until the end of the cohort study (December 2015). Endpoint definitions of the FRS, SCORE, and QRISK3 cohorts are summarized in Supplementary Table S6.

### Machine-learning algorithms

ML-based prediction models were developed to assess the participants’ 5-year risk for atherosclerotic CVD. Each cohort was partitioned into training/validation and test datasets in a 7:3 ratio using permutation. During the learning phase, the training/validation dataset was again divided into training and validation sets in an 8:2 ratio. The low overall event rate of CVD in the dataset posed the potential risk of biased predictions and misleading accuracy. Random oversampling was performed to develop a more balanced datasets during the training stage. We also obtained Cox-proportional hazard ratio to evaluate the association between 16 variables and endpoint in the PCE cohort (Supplementary Table S7). The predicted probability was a number between 0 and 1. Receiver operating characteristic curves were constructed, and the optimal cutoff value was determined by calculating Youden’s index for each model. Logistic regression and three other types of ML algorithms, including TreeBag, random forest, and neural networks, were pre-planned. One ML algorithm (AdaBoost) was added during the analysis. Logistic regression, which is also considered as an ML algorithm, uses a linear equation with independent predictors to predict a value^35^. TreeBag and random forest are algorithms that combine a multitude of decision trees via bagging^36, 37^. While random forest improves variance by reducing the correlation between trees, TreeBag uses random selection of variables for the best split at each node. AdaBoost combines weak learners into a weighted sum that represents the final output^38^. Neural networks are statistical learning algorithms mimicking the biological neuron system^39^. All ML algorithms were built using the R program. Supplementary Methods S1 section further elaborates on the machine learning techniques. The detailed architecture used in the neural networks is also described in Supplementary Methods and Supplementary Figure S1. The number of hidden layers and neurons in the layers were chosen empirically using the training/validation set. The consistency of the models was confirmed using fivefold cross-validation. The main models were constructed using the 16 baseline variables. A sensitivity analysis was done with models using the 8 variables that are commonly used in pre-existing prediction models.

### Statistical analysis

Analyses were performed separately in each cohort. Clinical characteristics are presented as numbers and percentages for categorical variables and means ± standard deviation for continuous variables. The performance of the contemporary and ML-based risk prediction models was assessed with respect to discrimination, calibration, and net benefit. Discrimination and calibration are the most commonly used parameters in risk prediction models. The overall performance was assessed using the Brier score, which was calculated as the squared differences between actual binary outcomes and predicted probabilities^40^. A lower score represented higher accuracy.

C-statistics and the 95% CIs were provided, to estimate the discrimination of the models. Delong’s test was used to compare two correlated C-statistics^41^. Predicted and observed event rates were compared for each model. Predictive accuracy, sensitivity, specificity, positive predictive values, negative predictive values, and F1 score were calculated, as shown below.

$${\text{Predictive accuracy}} = \frac{TP + TN}{{TP + FP + FN + TN}}$$

$${\text{Sensitivity}} = \frac{TP}{{TP + FN}}$$

$${\text{Specificity}} = { }\frac{TN}{{TN + FP}}$$

$${\text{Positive predictive value}} = \frac{TP}{{TP + FP}}$$

$${\text{Negative predictive value}} = \frac{TN}{{TN + FN}}$$

$${\text{F}}1{\text{ score}} = 2 \times \frac{{\left( {Senstivity \times Positive predictive value} \right)}}{{\left( {Senstivity + Positive predictive value} \right)}},$$

where TP indicates true positive, TN indicates true negative, FP indicates false positive and TN indicates true negative.

The goodness-of-fit (calibration) of the models was tested with the modified Hosmer–Lemeshow χ^2^ statistic^42^. Study subjects were divided into deciles based on their predicted risk. For pre-existing prediction models, the observed incidence rate per 1000 person-years was compared against the predicted 10-year risk in each cohort. Incidence rates per 1000 person-years were calculated by dividing the number of events that occurred during the follow-up period. Calibration of the ML-based algorithms and PCE was determined using the predicted and observed numbers of events at 5 years in the PCE cohort.

Decision-curve analysis was used to quantify the clinical usefulness of each prediction model in the PCE cohort^43^. A threshold probability indicates the relative weight of the harms of a false positive at which a patient would opt for treatment expecting its benefit. The net benefit of a model was calculated as the difference between the proportion of true positives and the proportion of false positives weighted by the odds of the selected threshold. Then net benefit was plotted across different threshold probabilities. A model that provides higher net benefit at a particular threshold is preferred. The net benefit was presented at cutoffs of 3.75% and 5%, which correspond to 7.5% and 10% thresholds, respectively, in blood cholesterol and high blood pressure guidelines^7, 8^.

Two-sided P values of less than 0.05 were considered statistically significant. All statistical analyses were performed with R programming version 3.3.3 (R Foundation for Statistical Computing, Vienna, Austria).

## Supplementary Information

Supplementary Information

## Abbreviations

ACC/AHA: American College of Cardiology /American Heart Association
AUC: Area under curve
CIs: Confidence intervals
CVD: Cardiovascular disease
FRS: Framingham risk score
ICD‐10: International Classification of Diseases, 10th Revision
MESA: Multi-Ethnic Study of Atherosclerosis
ML: Machine learning
NHIS-HEALS: National Health Insurance Service-Health Screening
PCE: Pooled cohort equation
SCORE: Systematic coronary risk evaluation
