# Supplementary material for: Pre-existing and machine learning-based models for cardiovascular risk prediction
Source: Sci Rep. 2021 Apr 26;11:8886. doi: 10.1038/s41598-021-88257-w (PMC8076166; doi:10.1038/s41598-021-88257-w)
Supplement: Supplementary file 1 — Supplementary Information [file 41598_2021_88257_MOESM1_ESM.pdf]

Pre-existing and machine learning-based models for cardiovascular risk prediction

Sang-Yeong Cho, MD[1], Sun-Hwa Kim, PhD[2], Si-Hyuck Kang, MD[2,3], Kyong Joon Lee, PhD[4], Dongjun Choi, MS[4], Seungjin Kang, MS[5], Sang Jun Park, MD[6], Tackeun Kim, MD[7], Chang-Hwan Yoon, MD, PhD[2,3], Tae-Jin Youn, MD, PhD[2,3], In-Ho Chae, MD, PhD[2,3]

[1]Department of Cardiology, Gyeongsang National University School of Medicine and Gyeongsang National University Changwon Hospital, Changwon, Korea; [2]Cardiovascular Center, Seoul National University Bundang Hospital, Seongnam-si, Korea; [3]Department of Internal Medicine, Seoul National University, Seoul, Korea; [4]Department of Radiology, Seoul National University Bundang Hospital, Seoul National University College of Medicine, Seongnam-si, Korea; [5]Office of eHealth Research and Businesses, Seoul National University Bundang Hospital, Seongnam-si, Korea; [6]Department of Ophthalmology, Seoul National University Bundang Hospital, Seoul National University College of Medicine, Seongnam-si, Korea; [7]Department of Neurosurgery, Seoul National University Bundang Hospital, Seoul National University College of Medicine, Seongnam-si, Korea.

Address for correspondence:

Si-Hyuck Kang, MD

Cardiovascular Center, Internal Medicine, Seoul National University Bundang Hospital

82, Gumi-Ro 173 Beon-Gil, Bundang-Gu, Seongnam-Si, Gyeonggi-Do, Korea, 13620

Telephone: 82-31-787-7027; Fax: 82-31-787-4290; E-mail: eandp303@snu.ac.kr

Supplementary Appendix

Supplementary Methods. Brief explanations of the machine learning algorithms, and detailed architecture of the neural network

Supplementary Figure 1. Neural network architecture

Supplementary Table 1. Observed event number and rates in each study cohort

Supplementary Table 2. Comparison of discrimination of machine-learning based novel risk prediction models in the test set of the pooled cohort equation cohort

Supplementary Table 3. Machine-learning based models in the test set of the Framingham risk score (FRS) cohort, Systematic COronary Risk Evaluation (SCORE) cohort, and QRISK3 cohort.

Supplementary Table 4. Brief comparison of established scoring systems

Supplementary Table 5. Operational definitions of baseline variables

Supplementary Table 6. Definitions of study endpoint in each cohort

Supplementary Table 7. Cox-proportional hazard ratio in the pooled cohort equation cohort

Supplementary Methods.

Brief explanations of the machine learning algorithms

Logistic regression was originally developed in the field of statistics but has become one of the most widely used machine learning techniques. Logistic regression is a classification algorithm based on probabilities. The model is basically similar with linear regression. However, the classifier output is transformed into a probability value using the logistic sigmoid function, which distributes between 0 and 1.

Random forests are an ensemble learning method for classification. A multitude of individual decision trees are constructed during the training phase, and they operate as an ensemble. Each individual tree makes a class prediction, and the mean prediction from the trees are averaged in the forest. Random decision forests prevent the decision trees' propensity to overfitting of the training set.

TreeBag is a bagging method for classification, which also uses decision trees. The bagging (bootstrap aggregating) algorithm is an ensemble algorithm used to improve model stability and accuracy of classification and regression trees. Bootstrapping creates a number of new bootstrap samples by drawing samples with replacement of the original training data. Multiple versions of a predictor are generated, and the aggregation averages the versions. Bagging helps to reduce variance and minimize overfitting.

AdaBoost (or adaptive boosting) is another ensemble classifier. Weak classifier algorithms (weak learners) are combined into a weighted sum to form a strong classifier. AdaBoost is adaptive in the sense that the algorithm is retrained iteratively by choosing the training set based on accuracy of the previous training. The weight of each trained classifier depends on the achieved accuracy. AdaBoost is sensitive to noisy data and outliers. However, it can be less susceptible to the overfitting problem than other machine learning algorithms.

Neural networks are computing systems that have been inspired by biological neural networks. A neural network algorithm is composed of a collection of connected units called artificial neurons. The output of each neuron is computed by non-linear function of the inputs. Multiple neurons in the input, output, and hidden layers are connected with each other. Each connection is assigned a weight that represents its relative importance. The weight increases or decreases the strength of the signal at a connection. Multiple types of neural networks have been developed, and the algorithms are increasingly used in many disciplines.

Detailed architecture of the neural network

The entire neural network consisted of three consecutive layers: the input layer, hidden layer, and output layer. The input layer contained 16 neurons, each of which corresponded to the 16 baseline variables. Next, the hidden layer comprised 8 hidden neurons, and each neuron had weighted connections with all 16 neurons in the input layer. The rectified linear unit (ReLU) function was attached to the end of each neuron in the hidden layer to determine its activation status. The output layer consisted of only two neurons, which were also fully connected to the 8 neurons in the previous hidden layer. The final probability of the 5-year risk for atherosclerotic CVD was computed by the 2-way softmax function.

In the training process, the optimal weights of the connections between the layers were estimated. We minimized cross-entropy loss between the computed probability and the observed event rates by the gradient descent algorithm. The initial network weight was randomly chosen to follow the standard normal distribution. L2 regularization was incorporated into the loss calculation to avoid overfitting. The learning rate started from 0.01 and decayed every 20,000 steps at a rate of 0.96.

Supplementary Figure 1. Neural network architecture

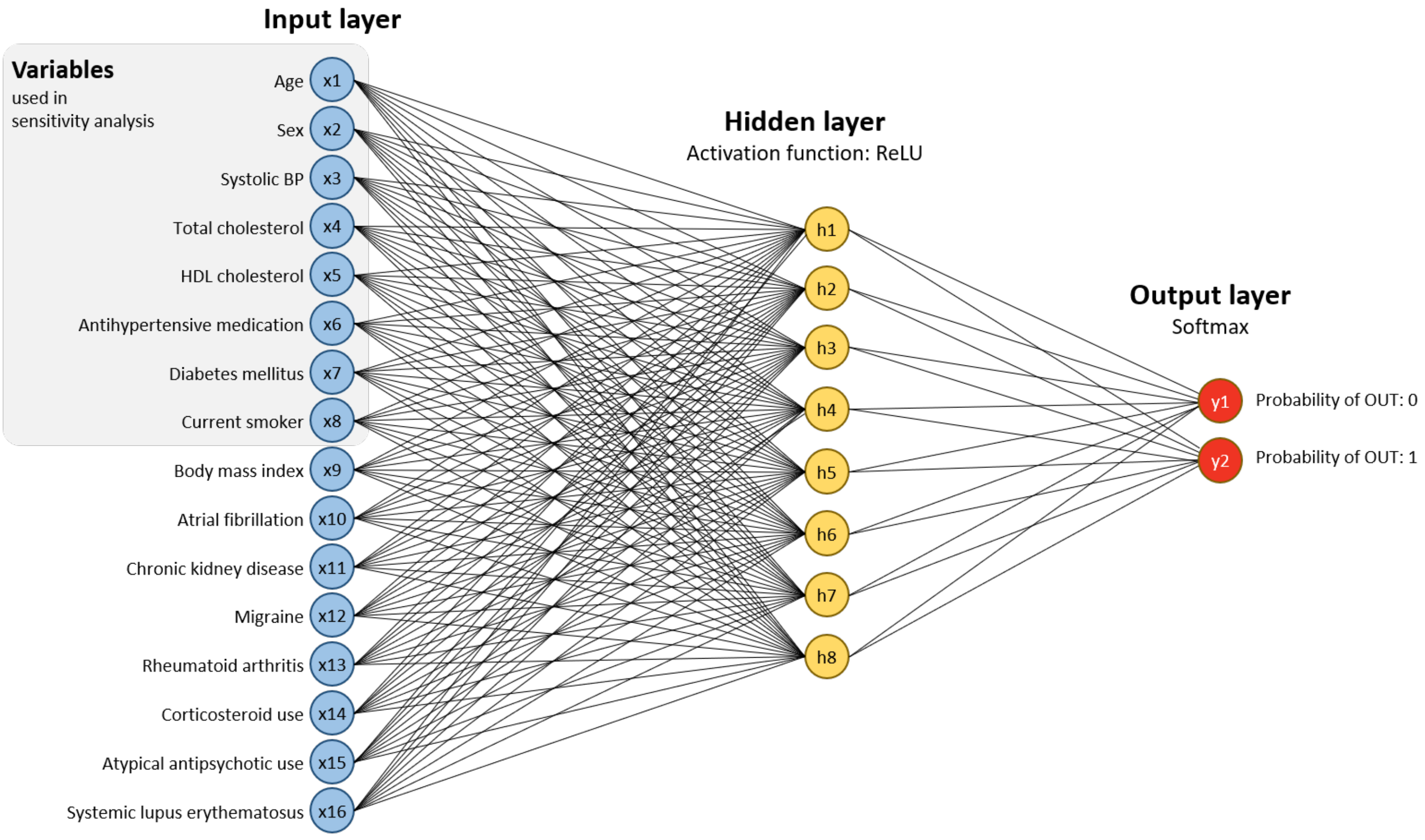

BP denotes blood pressure; HDL, high-density lipoprotein; ReLU, Rectified Linear Units

Supplementary Table 1. Observed event number and rates in each study cohort

**Characteristics**	**PCE cohort** (n=222,998)	**FRS cohort** (n=180,305)	**SCORE cohort** (n=166,824)	**QRISK cohort** (n=196,970)
Cardiac death	968 (0.43%)	584 (0.32%)	498 (0.30%)	355 (0.18%)
Angina		658 (0.36%)		831 (0.42%)
Acute myocardial infarction	1,961 (0.88%)	1,334 (0.74%)		1,765 (0.90%)
Stroke	5,501 (2.47%)	3,668 (2.03%)		
Ischemic stroke				3,518 (1.79%)
Transient ischemic attack				533 (0.27%)
Peripheral artery disease		264 (0.15%)		
Heart failure		1,142 (0.63%)		
Composite outcome	7,819 (3.51%)	6,206 (3.44%)	498 (0.30%)	5,777 (2.93%)

FRS denotes Framingham risk score; SCORE, Systematic COronary Risk Evaluation; PCE, pooled cohort equation.

Supplementary Table 2. Comparison of discrimination of machine-learning based risk prediction models in the test set of the pooled cohort equation cohort

								P values						
	Accuracy	Sensitivity	Specificity	PPV	NPV	F1 score	C-statistics (95% CI)	vs. PCE (A)	vs. PCE (W)	vs. LR	vs. RF	vs.TB	vs. AdaBoost	vs. NN
Pooled cohort equation (African)	65.8%	68.1%	65.7%	6.3%	98.4%	12.7%	0.726 (0.719–0.734)	-	-	-	-	-	-	-
Pooled cohort equation (white)	69.8%	67.1%	69.9%	7.0%	98.4%	12.6%	0.738 (0.727–0.749)	0.004	-	-	-	-	-	-
Logistic regression	67.8%	70.6%	67.7%	6.9%	98.6%	12.8%	0.749 (0.738–0.759)	<0.001	<0.001	-	-	-	-	-
Random forest	74.0%	59.0%	74.6%	7.2%	98.1%	9.7%	0.720 (0.709–0.731)	0.130	<0.001	<0.001	-	-	-	-
TreeBag	58.9%	67.2%	58.6%	5.2%	98.2%	11.9%	0.674 (0.662–0.685)	<0.001	<0.001	<0.001	<0.001	-	-	-
AdaBoost	65.0%	73.6%	64.7%	6.5%	98.6%	13.3%	0.740 (0.729–0.751)	<0.001	0.434	<0.001	<0.001	<0.001	-	-
Neural network (16 variables)	72.3%	65.6%	72.5%	7.4%	98.4%	12.9%	0.751 (0.740–0.761)	<0.001	<0.001	0.071	<0.001	<0.001	<0.001	-
Neural network (8 variables)	69.8%	68.9%	69.8%	7.1%	98.5%	12.7%	0.748 (0.738–0.759)	<0.001	<0.001	0.637	<0.001	<0.001	<0.001	0.051

PPV denotes positive predictive value; NPV, negative predictive value; CI, confidence intervals; PCE (A), pooled cohort equations for African-Americans; PCE (w), pooled cohort equations for whites; LR, logistic regression; RF, random forest; TB, treeBag; NN, neural network.

Supplementary Table 3. Machine-learning based models in the test set of the Framingham risk score (FRS) cohort, Systematic COronary Risk Evaluation (SCORE) cohort, and QRISK3 cohort.

	Accuracy	Sensitivity	Specificity	PPV	NPV	C-statistics (95% CI)	P values	
							vs. previous model	vs. LR
**FRS cohort**								
Framingham risk score	73.0%	57.2%	73.6%	6.8%	98.1%	0.704 (0.692–0.717)	-	-
Logistic regression	74.4%	58.5%	74.9%	7.3%	98.2%	0.725 (0.713–0.738)	<0.001	-
Neural network	73.5%	59.8%	74.0%	7.2%	98.2%	0.724 (0.712–0.737)	<0.001	0.748
**SCORE cohort**								
SCORE, low	70.1%	72.0%	70.1%	0.6%	99.9%	0.764 (0.720–0.808)	-	-
Logistic regression	82.1%	63.2%	82.2%	0.9%	99.9%	0.785 (0.741–0.828)	<0.001	-
Neural network	84.0%	59.2%	84.0%	0.9%	99.9%	0.650 (0.596–0.704)	0.083	<0.001
**QRISK cohort**								
QRISK3	69.4%	71.8%	69.3%	6.1%	98.9%	0.764 (0.752–0.776)	-	-
Logistic regression	74.1%	66.4%	74.3%	6.7%	98.8%	0.763 (0.751–0.776)	0.460	-
Neural network	69.2%	71.2%	69.1%	6.0%	98.9%	0.765 (0.753–0.777)	0.655	0.281

PPV denotes positive predictive value; NPV, negative predictive value; CI, confidence intervals; LR, logistic regression

Supplementary Table 4. Brief comparison of established scoring systems

	Pooled cohort equation	Framingham risk score	SCORE	QRISK3
Publication	2013 ACC/AHA guideline (Circulation 2013)	Agostino et al. (Circulation 2008)	Conroy et al. (Eur Heart J 2003)	Hippisley-Cox et al. (BMJ 2017)
N	24,262 (CARDIA, Framingham, ARIC, CHS)	8,491	205,178 (12 cohorts)	2,671,298
Age	Varying (40-79)	30-74 years	Varying (19-80)	25-84 years
CVD definition (Outcome)	Hard atherosclerotic cardiovascular disease<br>• Nonfatal myocardial infarction<br>• Coronary heart disease death<br>• Fatal or nonfatal stroke<br>•	• Coronary heart disease (coronary death, myocardial infarction, coronary insufficiency, and angina)<br>• Cerebrovascular events (ischemic stroke, hemorrhagic stroke, and transient ischemic accident)<br>• Peripheral artery disease (intermittent claudication)<br>• Heart failure	• Cardiovascular mortality (hypertension, acute coronary syndrome, ischemic heart disease, stroke, ventricular tachycardia, ventricular fibrillation, revascularization, atrioventricular block, peripheral artery disease, heart failure, etc)	• Coronary heart disease (angina and myocardial infarction)<br>• Stroke (hemorrhagic stroke excluded)<br>• Transient ischemic accident
Exclusion	• Clinical atherosclerotic cardiovascular disease (heart failure, ischemic heart disease, history of coronary artery bypass graft or percutaneous coronary intervention)<br>• Previous statin use<br>• Atrial fibrillation	• Coronary heart disease (coronary death, myocardial infarction, coronary insufficiency, and angina)<br>• Cerebrovascular events (ischemic stroke, hemorrhagic stroke, and transient ischemic accident)<br>• Peripheral artery disease (intermittent claudication)<br>• Heart failure	• Cardiovascular disease: previous MI, ACS, coronary revascularization (PCI or CABG), ischaemic stroke, PAD, chronic kidney disease<br>• Diabetes mellitus	• No recorded Townsend scores<br>• Cardiovascular disease<br>• Previous statin use

Supplementary Table 5. Operational definitions of baseline variables

Variables	Definition
Migraine	ICD-10 codes: G43.x and G44.x
Rheumatic arthritis	ICD-10 codes: M05.x
Use of steroid	Use of steroid tablet duration above 3 months (triamcinolone, prednisolone, betamethasone, hydrocortisone, dexamethasone, prednisone, budesonide, methylprednisolone)
Use of atypical antipsychotic drugs	Sum of pill medication duration above 1 months (amisulpride, aripiprazole, clozapine, lurasidone, olanzapine, paliperidone, quetiapine, risperidone, sertindole, or zotepine)
Systemic lupus erythematous	ICD-10 codes: M32.x
Atrial fibrillation	ICD-10 codes: I48.x
Chronic kidney disease	Estimated calculator CKD-EPI less than 60
Smoking status	Using self-report questionnaires: divided into 5 category groups, non-smoker, ex-smoker, light smoker (<10 cigarettes/day), moderate smoker (10-19 cigarettes/day), and heavy smoker (≥20 cigarettes/day).

Supplementary Table 6. Definitions of study endpoint in each cohort

Cohort	Endpoint	Definition
PCE cohort	Cardiovascular mortality	Death due to cardiovascular etiology (ICD-10 codes, I20-I25)
	Coronary heart disease	Discharge diagnosis (ICD-10 codes, I20 –I25) with coronary revascularization
	Cerebrovascular events	Discharge diagnosis (ICD-10 codes, I60–I64 and S64–S66) with brain imaging
FRS cohort	Coronary heart disease	Discharge diagnosis (ICD-10 codes, I20 –I25) with coronary revascularization
	Cerebrovascular events	Discharge diagnosis (ICD-10 codes, I60–I64 and S64–S66) with brain imaging
	Peripheral artery disease	Discharge diagnosis (ICD-10 codes, I708, I709, I743, I744, I745, I771)
	Heart failure	Discharge diagnosis (ICD-10 code, I50)
SCORE cohort	Cardiovascular mortality	Death due to cardiovascular etiology (ICD-10 codes, Ixx)
QRISK3 cohort	Cardiovascular mortality	Death due to cardiovascular etiology (ICD-10 codes, I20-I25, I63, I64, and G45)
	Coronary heart disease	Discharge diagnosis (ICD-10 codes, I20 –I25) with coronary revascularization
	Stroke	Discharge diagnosis (ICD-10 codes, I61) with brain imaging
	Transient ischemic accident	Diagnosis (ICD-10 code, G45) with brain imaging

FRS denotes Framingham risk score, ICD-10, International Classification of Diseases, 10th Revision; SCORE, Systematic COronary Risk Evaluation;

Supplementary Table 7. Cox-proportional hazard ratio in the pooled cohort equation cohort

**Variables**	**Unadjusted HR**	95% CI		P-value
		Lower limit	Upper limit	
Age (1 years)	1.098	1.094	1.101	<0.001
Male	1.361	1.262	1.468	<0.001
Body mass index (kg/m2)	0.964	0.951	0.976	<0.001
Systolic blood pressure (10mmHg)	1.237	1.211	1.265	<0.001
Diabetes mellitus	2.463	2.218	2.736	<0.001
Hypertension medication	2.297	2.133	2.473	<0.001
Smoking	1.569	1.444	1.705	<0.001
Atrial fibrillation/atrial flutter	2.878	2.418	3.424	<0.001
Migraine	1.349	1.187	1.534	<0.001
Systemic lupus erythematous	0.584	0.082	4.147	0.591
Rheumatoid arthritis	2.672	2.036	3.506	<0.001
Atypical drug use	3.807	2.06	5.562	<0.001
Chronic kidney disease	2.258	2.045	2.493	<0.001
Total cholesterol (10mg/dl)	1.000	0.999	1.001	0.992
HDL-cholesterol (10mg/dl)	0.965	0.945	0.985	<0.001
Steroid Use	1.951	1.674	2.274	<0.001

CI denotes confidence intervals, HDL; high-density lipoprotein, HR; hazard ratio
